# Abcc6 deficiency in mice leads to altered ABC transporter gene expression in metabolic active tissues

**DOI:** 10.1186/s12944-018-0943-x

**Published:** 2019-01-05

**Authors:** Bettina Ibold, Isabel Faust, Janina Tiemann, Theo G. M. F. Gorgels, Arthur A. B. Bergen, Cornelius Knabbe, Doris Hendig

**Affiliations:** 1 Institut für Laboratoriums- und Transfusionsmedizin, Herz- und Diabeteszentrum Nordrhein-Westfalen, Universitätsklinik der Ruhr-Universität Bochum, Georgstraße 11, D-32545 Bad Oeynhausen, Germany; 2 University Eye Clinic Maastricht, Maastricht University Medical Center, 6202 AZ Maastricht, The Netherlands; 3 Netherlands Institute for Neurosciences (NIN-KNAW), Amsterdam, The Netherlands; 4 Academic Medical Centre, University of Amsterdam, 1100 DD Amsterdam, The Netherlands

**Keywords:** *Abcc6*, Pseudoxanthoma elasticum, Gene expression, ATP-binding cassette transporters, Mice

## Abstract

**Background:**

ATP-binding cassette (ABC) transporters are involved in a huge range of physiological processes. Mutations in the *ABCC6* gene cause pseudoxanthoma elasticum, a metabolic disease with progressive soft tissue calcification.

**Methods:**

The aim of the present study was to analyze gene expression levels of selected ABC transporters associated with cholesterol homeostasis in metabolic active tissues, such as the liver, kidney and white adipose tissue (WAT) of *Abcc6*^*−/−*^ mice from an early and late disease stage (six-month-old and 12-month-old mice).

**Results:**

The strongest regulation of ABC transporter genes was observed in the liver tissue of six-month-old *Abcc6*^*−/−*^ mice. Here, we found a significant increase of mRNA expression levels of phospholipid, bile salt and cholesterol/sterol transporters *Abcb1b*, *Abcb11*, *Abcg1, Abcg5* and *Abcg*8. *Abcd2* mRNA expression was increased by 3.2-fold in the liver tissue. We observed strong upregulation of *Abca3* and *Abca1* mRNA expression up to 3.3-fold in kidney and WAT, and a 2-fold increase of *Abca9* mRNA in the WAT of six-month-old *Abcc6* knockout mice. Gene expression levels of *Abcb1b* and *Abcg1* remained increased in the liver tissue after an age-related disease progression, while we observed lower mRNA expression of *Abca3* and *Abca9* in the kidney and WAT of 12-month-old *Abcc6*^*−/−*^ mice.

**Conclusions:**

These data support previous findings that Abcc6 deficiency leads to an altered gene expression of other ABC transporters depending on the status of disease progression. The increased expression of fatty acid, bile salt and cholesterol/sterol transporters may be linked to an altered cholesterol and lipoprotein metabolism due to a loss of Abcc6 function.

**Electronic supplementary material:**

The online version of this article (10.1186/s12944-018-0943-x) contains supplementary material, which is available to authorized users.

## Background

Eukaryotic members of the ATP-binding cassette (ABC) superfamily mediate the export of a wide range of substrates and have, therefore, a functional importance in various physiological processes. These transporter proteins use ATP hydrolysis to move the respective substrate against their concentration gradient. The ABC transporters are grouped into seven subfamilies depending on structural similarities and conserved sequence motifs. There is a high sequence homology between ABC transporters of mice and man (summarized in [1]). The ABC transporters are highly variable expressed in different tissues. High transporter expression was found in tissues involved in secretory, reproductive and metabolic functions, such as the liver and kidney [2]. Twenty-two of the 48 human ABC transporters have been implicated in causing monogenetic diseases, such as Tangier disease (gene: *ABCA1*) and Dubin-Johnson syndrome (gene: *ABCC2*) [1]. Mutation analyses demonstrated that alterations of *ABCC6* gene sequence cause pseudoxanthoma elasticum (PXE; OMIM 264800) [3, 4], an autosomal-recessive disease, which is characterized by progressive calcification of connective tissue and manifests in early adolescence. Patients with PXE suffer from skin lesions and ocular manifestations which are associated with spontaneous subretinal neovascularization and hemorrhage. Mineralization of the internal elastic lamina of blood vessels can lead to cardiovascular complications [5]. The level of *ABCC6* expression is high in liver and kidney, and much lower or absent in the tissues affected. Recent studies suggested that ABCC6 acts in cellular and systemic pyrophosphate homeostasis although the physiological/endogenous substrates remain unknown [6, 7]. *Abcc6* knockout (*Abcc6*^−/−^) mice develop a PXE-like phenotype and are, therefore, a suitable model for studying the genesis of PXE. Ectopic mineralization of blood vessels first occurs in six-month-old *Abcc6*^−/−^ mice in many tissues and progresses with age [8].

In this study, we have examined the expression profile of selected ABC transporter genes associated with cholesterol homeostasis [9–11] in *Abcc6*^−/−^ mice compared to wild type (WT) mice, because there are several indications that PXE is linked to an alteration of cholesterol metabolism accompanied by altered ABC transporter gene expression [8, 12, 13]. We analyzed gene expression profiles in tissues from metabolic active sites, such as the liver, kidney and white adipose tissue (WAT) [2, 14] from 6- and 12-month-old mice as these ages in mice recapitulate age at disease onset and progressed disease manifestation in PXE patients.

## Materials and methods

### Animals

All animal preparations comply with law on animal welfare of Germany used for scientific purposes. Therefore, an ethical approval is not required. Mice were killed solely for the use of their organs or tissues without prior burden. The *Abcc6*^*−/−*^ mice were generated on a hybrid background of C57BL/6 and 129/Ola and backcrossed to C57BL/6 [8]. Mice were housed in the central animal facility of Bielefeld University (Germany) and kept with water and food (normal chow) ad libitum. In the present study, we used *Abcc6* (+/+) littermates and pure C57BL/6 mice as WT control mice. Mice (males and females), aged 6 months ± 2 weeks and 12 months ± 4 weeks, were anesthetized, intraperitoneal with 0.65 mg ketamine, 0.02 mg acepromazine and 0.13 mg xylazine per 10 g bodyweight and sacrificed by cervical dislocation. After opening the thorax, the right ventricle of the heart was cut for liver perfusion via the hepatic portal vein with phosphate-buffered saline buffer (PBS). The other tissues were only washed in PBS. Liver, kidney and visceral WAT (gonadal) were collected, frozen immediately in liquid N_2_ and stored at − 80 °C until use.

### RNA extraction from tissue and cDNA synthesis

Total RNAs were extracted from 100 mg liver, kidney and visceral WAT (gonadal) using QIAzol reagent (Qiagen, Hilden, Germany), followed by a purification using RNeasy Mini protocol (Qiagen, Hilden, Germany). Total RNA was treated with DNase I (Macherey-Nagel™, Bottrop, Germany) on mini-columns to eliminate genomic DNA. The RNA quantification was assessed by using the NanoDrop 2000 spectrophotometer (Thermo Fisher, Schwerte, Germany) and RNA quality was determined using the Agilent RNA 6000 Nano Kit (Agilent Technologies, Ratingen, Germany), according to the manufacturer’s instructions.

First-strand cDNA was synthesized from 1 μg of total RNA for each reaction using the SuperScript II Reverse Transcriptase Kit (Thermo Fisher, Schwerte, Germany), according to the manufacturer’s instructions. The cDNA was diluted 1:5 or 1:10 with water, depending on the target gene, and stored at − 20 °C prior to quantitative real-time PCR (qRT-PCR).

### Quantitative real-time PCR

The qRT-PCR was performed on a LightCycler480 (Roche, Mannheim, Germany) using Lightcycler480 MasterCycler SYBR® Green (Roche, Mannheim, Germany) to assess the mRNA expression levels of target and reference genes. All intron-spanning primers used for qRT-PCR analysis were designed with Clone Manager Suite 7 (Scientific & Educational Software), synthesized by Biomers (Ulm, Germany) and are listed in the Additional file 1: Table S1. The PCR thermal cycling conditions contained an initial incubation of 5 min at 95 °C, followed by 45 cycles of 10 s degradation at 95 °C, primer-specific annealing for 15 s at 65 or 59 °C, and 20 s elongation and detection of the amplicon at 72 °C. Finally, a melting curve analysis of the amplicon was performed. Each cDNA sample was run in technical triplicates. Water was used as a negative control for each primer pair. The relative amount of target mRNA in each sample was calculated using the ΔΔCt method, as previously described [15]. Relative mRNA expression levels were corrected by PCR efficiency and the reference genes normalization factor, by normalizing target mRNA Ct values to those of glyceraldehyde-3-phosphate dehydrogenase (*Gapdh*), hypoxanthine phosphor-ribosyltransferase 1 (*Hprt*) and beta-2 microglobulin (*ß2m*) (6-month-old mice) or *Gapdh, Hprt* and eukaryotic translation initiation factor 3 subunit A (*Eif3a*) (12-month-old mice). A cutoff for no detectable mRNA expression was set to a Ct value of 35.

### Statistical analysis

Data are presented in arbitrary units as means with corresponding standard error (SEM). Graphic data processing and statistical analysis were performed with GraphPad Prism 5 (GraphPad Software, Inc.), using Student’s t-tests for two group comparisons and the non-parametric Mann-Whitney U test for data which are not Gaussian distributed. The Shapiro-Wilk test was used to determine whether data are normally distributed or not. Significance was accepted at *p* ≤ 0.05 (two-tailed).

## Results

The mRNA expression profiles were compared between *Abcc6*^*−/−*^ mice and age-matched WT mice to examine the effects of Abcc6 deficiency on gene expression levels of other ABC transporters. We determined the relative gene expression of selected ABC subfamily genes (out of subfamilies a, b, c, d and g) in the liver, kidney and visceral WAT (gonadal) of 6- and 12-month-old *Abcc6*^−/−^ and WT mice by qRT-PCR.

### ABC transporter gene expression in the liver, kidney and white adipose tissue of 6-month-old *Abcc6*^*−/−*^ mice

**Fig. 1:**
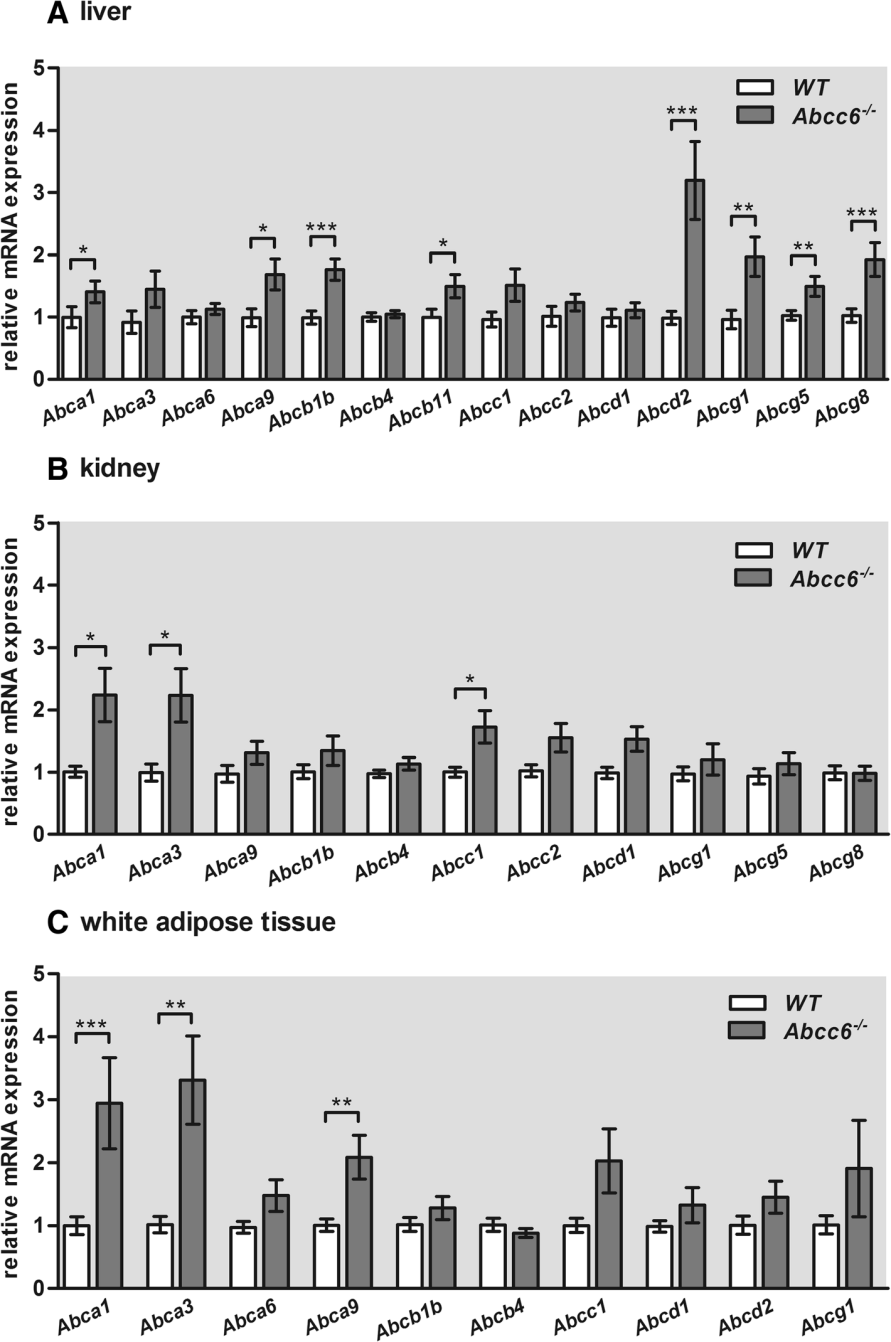
ABC transporter gene expression in liver, kidney and white adipose tissue of 6-month-old *Abcc6*^*−/−*^ mice. Normalized relative gene expression level of various ABC transporters in the (**a**) liver (**b**) kidney and (**c**) WAT of 6-month-old ± 2 weeks male and female WT (liver: *n* = 25; kidney: n = 25; WAT: *n* = 21, respectively) and *Abcc6*^−/−^ mice (liver: *n* = 22; kidney: *n* = 23; WAT: *n* = 18, respectively) by qRT-PCR. Relative mRNA expression of ABC transporter members belonging to a, b, c, d and g subfamily. Data are presented as fold change of mean ± SEM relative to samples of age-matched WT. Statistically significant differences were analyzed using unpaired, two-tailed Student’s t-test and are indicated in the following manner: * *p* ≤ 0.05; ** *p* ≤ 0.01; *** *p* ≤ 0.001

Alterations of mRNA expression of ABC transporters were most pronounced in the liver tissue of 6-month-old *Abcc6*^*−/−*^ mice compared to WT mice (Fig. 1a). *Abcc6*^−/−^ mice showed an *Abca9* mRNA level increase of 70%. The relative mRNA expression of *Abcb1b* and *Abcb11* were 1.8, respectively, 1.5 times higher in *Abcc6*^*−/−*^ mice. The hepatic transcript levels of transporters of the Abcg subfamily were significantly higher in *Abcc6*^*−/−*^ mice relative to WT mice. In comparison to WT mice, relative gene expression of *Abcg1* was 2-fold, that of *Abcg5* was 1.5-fold and that of *Abcg8* was 1.9-fold increased in Abcc6-deficient mice. The mRNA levels of *Abcd2* were also significantly upregulated (3.2-fold) in *Abcc6*^*−/−*^ mice.

Absence of functional *Abcc6* also affected the mRNA expression of ABC transporter genes in the kidney and WAT of 6-month-old mice. However, relative gene expression of the *Abca3* transporter was significantly increased (2.2-fold) in kidney tissue and in the WAT (3.3-fold) of *Abcc6*^−/−^ mice compared to age-matched WT mice (Fig. 1b, c). Figure 1b shows that the relative mRNA expression of *Abca1* (2.2-fold) and *Abcc1* (1.7-fold) were significantly increased in kidney tissue of 6-month-old *Abcc6*^*−/−*^ mice. We also revealed a significantly higher level of mRNA of the *Abca1* gene (3-fold) and the *Abca9* gene (2-fold) in the WAT of Abcc6-deficient mice relative to WT mice (Fig. 1c). Relative mRNA expression levels of the other ABC transporter genes analyzed did not significantly change between Abcc6-deficient and WT mice.

### ABC transporter gene expression in the liver, kidney and white adipose tissue of 12-month-old *Abcc6*^*−/−*^ mice

**Fig. 2:**
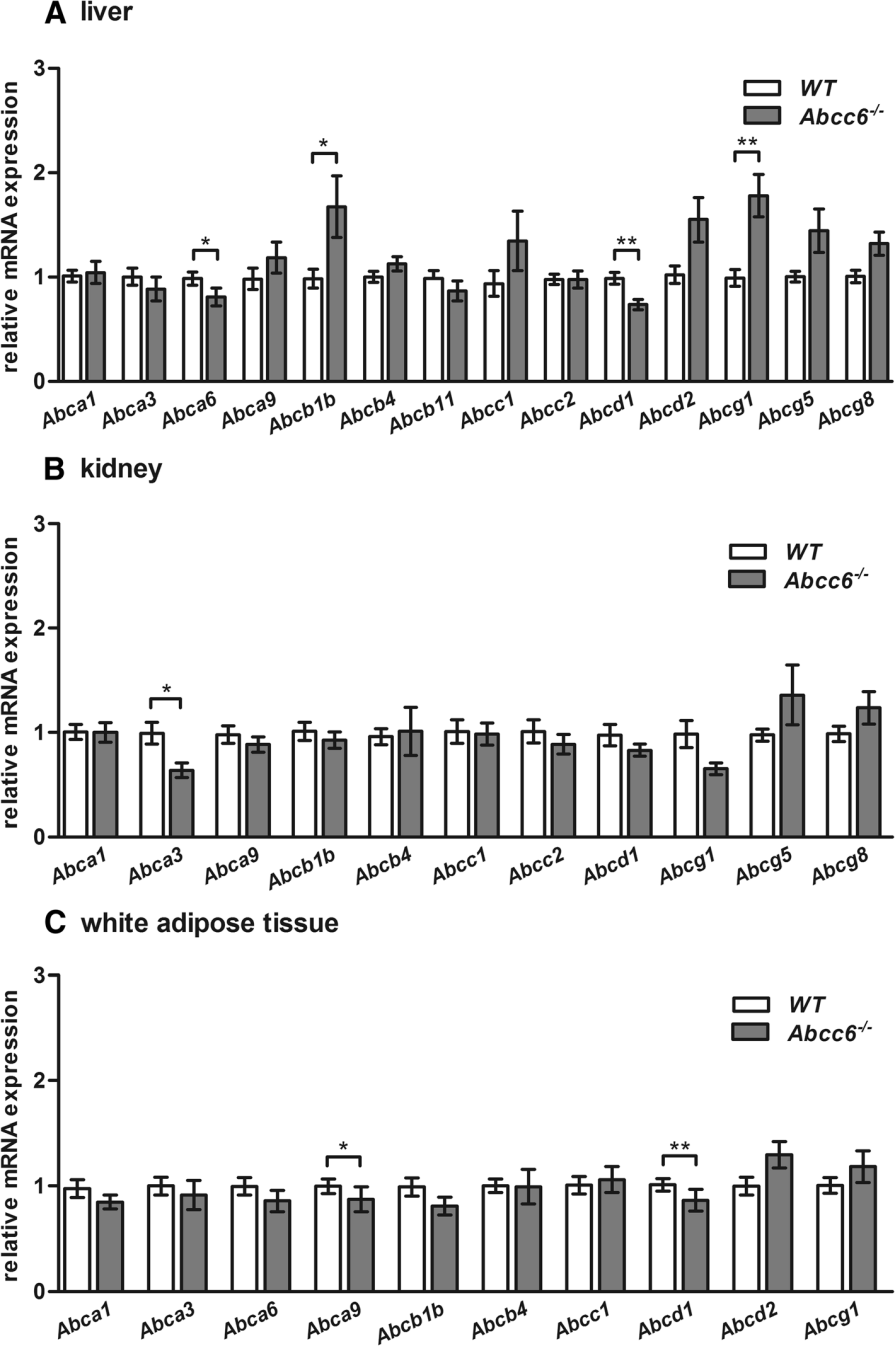
ABC transporter gene expression in liver, kidney and white adipose tissue of 12-month-old *Abcc6*^*−/−*^ mice. Normalized relative gene expression level of various ABC transporters in the (**a**) liver (**b**) kidney and (**c**) WAT of 12-month-old ± 4 weeks male and female WT (liver: *n* = 37; kidney: *n* = 34; WAT: *n* = 33, respectively) and *Abcc6*^−/−^ mice (liver: *n* = 39; kidney: *n* = 42; WAT: *n* = 38, respectively) by qRT-PCR. Relative mRNA expression of ABC transporter members belonging to a, b, c, d and g subfamily. Data are presented as fold change of mean ± SEM relative to samples of age-matched WT. Statistically significant differences were analyzed using unpaired, two-tailed Student’s t-test and are indicated in the following manner: * *p* ≤ 0.05; ** *p* ≤ 0.01; *** *p* ≤ 0.001

We found that the mRNA expression levels of members of the ABC transporter family a and c (*Abca1, Abca3, Abca9, Abcc1* and *Abcc2*) in the liver tissue of 12-month-old mice were almost comparable to those found in WT mice (Fig. 2a). Post hoc analysis revealed that the *Abca6* mRNA down-regulation (0.8-fold) was significant in Abcc6-deficient mice compared to WT mice. Additionally, a statistically insignificant increase in *Abcb1b* expression (1.7-fold) in the liver of *Abcc6*^*−/−*^ mice was detected. Hepatic *Abcd1* mRNA levels were decreased by 26% in *Abcc6*^−/−^ mice in comparison to WT mice. However, we identified an mRNA level increase of all Abcg transporter family member genes (*Abcg1, Abcg5* and *Abcg8*) measured in the liver tissue of *Abcc6*^−/−^ mice. *Abcg1* mRNA expression was significantly upregulated (1.8-fold) in *Abcc6*^−/−^ mice.

We found significantly lower mRNA expression levels of *Abca3* (0.6-fold) in the kidney tissue of 12-month-old Abcc6-deficient mice in comparison to WT mice (Fig. 2b). No significant difference in the gene expression of *Abca1* and *Abcc1* was observed.

Gene expression of almost all selected ABC transporters in the WAT of 12-month-old mice was lower in *Abcc6*^−/−^ mice (Fig. 2c). The mRNA expression levels of *Abca9* and *Abcd1* (0.8-fold each) were significantly lower in the WAT of Abcc6-deficient mice compared to WT mice.

## Discussion

Recent studies demonstrated that patients with Dubin-Johnson syndrome and *Abcc2* knockout rats expressed the *Abcc3* gene at a higher level in comparison to WT rats [16]. Compensatory gene expression of *Abcd2* was also found in *Abcd1*^*−/−*^ mice [17]. In the case of Abcc6 deficiency, Li et al. observed a compensatory, ~ 6.5-fold increase of *Abca4* gene expression in the liver of 1-month-old Abcc6-deficient mice, but not in kidney and eyes [18]. We showed in our previous study that a loss of ABCC6 function in human dermal fibroblasts results in a compensatory upregulated expression of several ABC transporters, such as *ABCA6* and *ABCA9,* whereas *ABCA3* was decreased [13]. PXE is described as a systemic disease [19] and ABCC6 expression levels are at their highest in metabolically active tissues, such as liver and kidney [20]. Consequently, we expected strong compensatory changes in the expression profiles of ABC transporters in these tissues.

We observed in the liver tissue of 6-month-old *Abcc6*^−/−^ mice that mRNA levels of *Abcd2* and the cholesterol transporter gene *Abcg1* as well as mRNA levels of phospholipid, bile salt and cholesterol/sterol transporters *Abcb1b*, *Abcb11*, *Abcg5* and *Abcg8*, respectively, were significantly upregulated relative to WT mice. By contrast, analysis of phospholipid, bile salt and sterol transporter gene expression in the kidney and WAT of 6- and 12-month-old *Abcc6*^−/−^ mice revealed no differences compared to WT mice.

Gorgels et al. reported that *Abcc6*^*−/−*^ mice developed a 25% reduction in plasma HDL cholesterol and total cholesterol [8]. The increase of *Abca1* mRNA levels in the liver, kidney and WAT of 6-month-old *Abcc6*^*−/−*^ mice and the upregulation of the hepatic *Abcg1* gene expression in 6- and 12-month-old *Abcc6*^*−/−*^ mice may be associated with the change of HDL cholesterol levels. Tangier disease due to ABCA1 deficiency is associated with very low HDL plasma levels and an upregulation of *ABCG1* gene expression [21]. The cholesterol depletion proposed might also lead to an induction of *Abcd2* mRNA expression, which has already been shown for fibroblasts and monocytes via an activation of transcription factors belonging to the sterol regulatory element-binding protein family [22]. The ABCD transporter family is responsible for the transport of very long-chain fatty acids over the peroxisomal membrane, where they are degraded by ß-oxidation [23]. Up-regulation of *Abcd2* mRNA expression in *Abcc6*^*−/−*^ mice might accompany induced ß-oxidation. We have previously shown increased fatty acid oxidation in skin fibroblasts derived from PXE patients [24].

We showed that gene expression levels of *Abcc1* and *Abcc2* in the liver of 6- and 12-month-old *Abcc6*^*−/−*^ mice were unaltered compared to WT mice. This observation is in accordance with a previous study analyzing 1-month-old *Abcc6* knockout mice [18].

In addition, other members of Abca subfamily seem to be affected by Abcc6 deficiency [13, 18]. Significant alterations of *Abca3* mRNA levels in the kidney and WAT of 6-month-old and in the kidney of 12-month-old mice were detected. The Abca3 protein is not only highly abundant in lamellar bodies, a storage organelle enriched in mixture phospholipids, neutral lipids, cholesterol and hydrophobic proteins, of the lung, but also detectable in extrapulmonary tissues, such as the kidney and liver [25]. *Abca3* expression is decreased after ischemic reperfusion injury in mouse kidney [26], but the role of Abca3 in lipid distribution of extrapulmonary tissues and organs remains unclear. The *Abca9* gene has a very high sequence homology to *Abca8* (72%) [10]. An increase of *Abca8* expression levels is associated with the induction of reverse cholesterol transport mediated by HDL particles [27]. It is possible that changes in *Abca9* expression levels in the WAT of 6- and 12-month-old mice reflect aberrations of the HDL metabolism of *Abcc6*^−/−^ mice.

We decided to analyze two different ages of *Abcc6*^*−/−*^ mice, as PXE is a progressive disorder, and, therefore, expected changes in gene expression profiles. Moreover, 6- and 12-month-old mice are comparable in age to patients at an early and at a late disease stage of PXE disease. It has already been shown that ABC transporter expression profiles depend on disease states [28], which might explain the different mRNA expression profiles of ABC transporters in tissues from 6- and 12-month-old *Abcc6*^*−/−*^ mice.

## Conclusions

In summary, Abcc6 deficiency in mice causes the aberrant gene expression of ABC transporters involved in cholesterol metabolism. These findings reinforce our hypothesis that ABCC6 has a functional role in lipoprotein and cholesterol homeostasis [12], especially in HDL cholesterol. However, the underlying molecular mechanisms behind these changes remain unknown and must be investigated in future studies. It is noteworthy that protein expression of the investigated genes was not analyzed due to study limitations. Therefore, protein expression may differ from mRNA expression.

## Additional file

Additional file 1: **Table S1.** Primer sequences used for qPCR (DOCX 25 kb)

## Abbreviations

Abc: ATP-binding cassette
ATP: Adenosine triphosphate
PXE: Pseudoxanthoma elasticum
WAT: White adipose tissue
WT: Wildtype
