# Supplementary material for: Abcc6 deficiency in mice leads to altered ABC transporter gene expression in metabolic active tissues
Source: Lipids Health Dis. 2019 Jan 5;18:2. doi: 10.1186/s12944-018-0943-x (PMC6320597; doi:10.1186/s12944-018-0943-x)
Supplement: Supplementary file 1 — Table S1. Primer sequences used for qPCR (DOCX 25 kb) [file 12944_2018_943_MOESM1_ESM.docx]

**Suppl. Table 1.**  Primer sequences used for qPCR

| **Target gene** | **Sequence 5´- 3´** | **Reference sequence^A^** | **Product size (bp)** | **Annealing (°C)^B^** | **Melting (°C)^C^** |  |
| --- | --- | --- | --- | --- | --- | --- |
| ***Actb***  *Actin, beta* | TGCTGTCCCTGTATGCCTCT  AGGTCTTTACGGATGTCAACG | NM_007393.4 | 463 | 59 | 88.0 |  |
| ***Gapdh***  *glyceraldehyde-3-phosphate dehydrogenase* | GCATCTTGGGCTACACTGAGG  GGGTGGTCCAGGGTTTCTTAC | NM_008084.3 | 211 | 59 | 85.4 |  |
| ***ß2m***  *beta-2 microglobulin* | GGTCTTTCTGGTGCTTGTCTC  GCAGGCGTATGTATCAGTCTC | NM_009735.3 | 280 | 59 | 83.5 |  |
| ***Eif3a***  *Eukaryotic translation initiation factor 3 subunit A* | GAGTATCAGGAGCGAGTCAAG  CCTCTCATCATCCCGAGTTTC | NM_010123.3 | 255 | 59 | 83.6 |  |
| ***Abca1***  *ATP-binding cassette, sub-family A, member 1* | ATAAAGCCATGCCGTCTG  TGCTTGATCTGCCGTAAC | NM_013454.3 | 238 | 59 | 85.3 |  |
| ***Abca3***  *ATP-binding cassette, sub-family A, member 3* | CTCTTGTCCTGTCTCCTATC  GTCCTTATTGCCCACTTG | NM_013855.3 | 423 | 59 | 87.9 |  |
| ***Abca6***  *ATP-binding cassette, sub-family A, member 6* | AGCCTGGAGTGACCACATC  CCTCCGCCATTTCTTGAGC | NM_147218.2 | 250 | 65 | 85.4 |  |
| ***Abca9***  *ATP-binding cassette, sub-family A, member 9* | CCCTCCTTTCACACTGATTGG  TTCGGAACAGCAGGACAAC | NM_147220.2 | 237 | 65 | 80.7 |  |
| ***Abcb1b***  *ATP-binding cassette, sub-family B, member 1b* | GGCGTATTTGGGATGTTTCG  CCTGGATGTAGGCAACTATGAG | NM_011075.2 | 295 | 65 | 82.7 |  |
| ***Abcb4***  *ATP-binding cassette, sub-family B, member 4* | GTGTCCCAGGAACCCATTCTC  GTGGCTTCATCCAGCAGTAGG | NM_008830.2 | 263 | 63 | 86.9 |  |
| ***Abcb11***  *ATP-binding cassette, sub-family B, member 11* | GGAAGAGCACAGCACTAC  CCAGAAGCAGGATCTTGG | NM_021022.3 | 369 | 63 | 85.2 |  |
| ***Abcc1***  *ATP-binding cassette, sub-family C, member 1* | TGGCATCACCTTCTCCATTC  GGTGCCCAAACAGTATGTTC | NM_008576.3 | 206 | 59 | 88.7 |  |
| ***Abcc2***  *ATP-binding cassette, sub-family C, member 2* | GGTGTTGGGCTTATGGTTC  GCAGCAAGTTCCTGAGTTC | NM_013806.2 | 217 | 59 | 80.5 |  |
| **Target gene** | **Sequence 5´- 3´** | **Reference sequence^A^** | **Product size (bp)** | **Annealing (°C)^B^** | **Melting (°C)^C^** |  |
| ***Abcd1***  *ATP-binding cassette, sub-family D, member 1* | CAATGGCTGTGGCAAGAG  TGGAGAGCAGTGCGATAC | NM_007435.2 | 446 | 59 | 88.1 |  |
| ***Abcd2***  *ATP-binding cassette, sub-family D, member 2* | GCTTCCAGGCTAAACTTC  GGCACTGGTACATTCATC | NM_011994.2 | 435 | 63 | 84.1 |  |
| ***Abcg1***  *ATP-binding cassette, sub-family G, member 1* | AACGTGGATGAGGTTGAG  TTCCCAGAGATCCCTTTC | NM_009593.2 | 203 | 59 | 86.0 |  |
| ***Abcg5***  *ATP-binding cassette, sub-family G, member 5* | CTGCTGAGGCGAGTAACAAG  AATGACCGTGGCGATGAC | NM_031884.2 | 333 | 59 | 87.5 |  |
| ***Abcg8***  *ATP-binding cassette, sub-family G, member 8* | AATGCCCTCTACAACTCC  CTGATGCCGATGACAATG | NM_026180.3 | 263 | 59 | 84.4 |  |

A Reference sequence taken from GenBank. Accession numbers are presented (2017).

B Annealing temperature in °C.

C Melting temperature of the amplicon in °C.
